# Selective knockdown of hexokinase 2 in rods leads to age-related photoreceptor degeneration and retinal metabolic remodeling

**DOI:** 10.1038/s41419-020-03103-7

**Published:** 2020-10-20

**Authors:** Rui Zhang, Weiyong Shen, Jianhai Du, Mark C. Gillies

**Affiliations:** 1 Save Sight Institute, Discipline of Ophthalmology, Sydney Medical School, University of Sydney, Sydney, NSW 2000 Australia; 2 Department of Ophthalmology, West Virginia University, Morgantown, WV 26506 USA; 3 Department of Biochemistry, West Virginia University, Morgantown, WV 26506 USA

**Keywords:** Molecular neuroscience, Neurological disorders

## Abstract

Photoreceptors, the primary site of phototransduction in the retina, require energy and metabolites to constantly renew their outer segments. They preferentially consume most glucose through aerobic glycolysis despite possessing abundant mitochondria and enzymes for oxidative phosphorylation (OXPHOS). Exactly how photoreceptors balance aerobic glycolysis and mitochondrial OXPHOS to regulate their survival is still unclear. We crossed rhodopsin-Cre mice with hexokinase 2 (HK2)-floxed mice to study the effect of knocking down HK2, the first rate-limiting enzyme in glycolysis, on retinal health and metabolic remodeling. Immunohistochemistry and Western blots were performed to study changes in photoreceptor-specific proteins and key enzymes in glycolysis and the tricarboxylic acid (TCA) cycle. Changes in retinal structure and function were studied by optical coherence tomography and electroretinography. Mass spectrometry was performed to profile changes in ^13^C-glucose-derived metabolites in glycolysis and the TCA cycle. We found that knocking down HK2 in rods led to age-related photoreceptor degeneration, evidenced by reduced expression of photoreceptor-specific proteins, age-related reductions of the outer nuclear layer, photoreceptor inner and outer segments and impaired electroretinographic responses. Loss of HK2 in rods led to upregulation of HK1, phosphorylation of pyruvate kinase muscle isozyme 2, mitochondrial stress proteins and enzymes in the TCA cycle. Mass spectrometry found that the deletion of HK2 in rods resulted in accumulation of ^13^C-glucose along with decreased pyruvate and increased metabolites in the TCA cycle. Our data suggest that HK2-mediated aerobic glycolysis is indispensable for the maintenance of photoreceptor structure and function and that long-term inhibition of glycolysis leads to photoreceptor degeneration.

## Introduction

Photoreceptors, the primary site of phototransduction in the retina, require high energy and abundant metabolites to synthesize proteins and lipids for constant renewal of their outer segments^1–3^. They shed around 10% of their outer segments every day, resulting in full renewal of the outer segments every 10 days^1,4^. Photoreceptor degeneration, including loss of photoreceptor outer segments (POS), is found in many retinal diseases including retinal detachment^5^, inherited retinal degenerations^6^, age-related macular degeneration^7^, diabetic macular edema^8^, and macular telangiectasia type 2^9^. Retinal metabolic dysfunction is a potential cause of photoreceptor degeneration^7,10–14^.

Photoreceptors preferentially metabolize glucose through aerobic glycolysis (the “Warburg Effect”) to meet their energetic and anabolic demand, although they have abundant mitochondria for oxidative phosphorylation (OXPHOS)^15–18^. Hexokinase (HK), the first rate-limiting enzyme in glycolysis, has four isozymes. HK1 is found in all mammalian tissues and considered as a “housekeeping enzyme” in physiological conditions. HK2 is the isoform that is highly expressed in photoreceptors^15,19^. HK1 and HK2 can associate with voltage-dependent-anion channel (VDAC) to regulate mitochondrial function^20,21^. Little is known about the expression and function of HK3 and HK4 in the retina. Among the four isoforms of HK enzymes, HK2 plays a key role in the Warburg effect exhibited by photoreceptors^15,19^. Photoreceptors also express other key enzymes of glycolysis and the tricarboxylic acid (TCA) cycle, including pyruvate kinase and pyruvate dehydrogenase^13,15,22,23^. Pyruvate kinase muscle isozyme 2 (PKM2), highly expressed in photoreceptors, is responsible for converting phosphoenolpyruvate to pyruvate at the last step of glycolysis. Pyruvate is converted to lactate through lactate dehydrogenase A (LDH-A) or to acetyl-CoA through pyruvate dehydrogenase complex. Pyruvate dehydrogenase E1 subunit alpha (PDHE1α) is a key element in the pyruvate dehydrogenase complex^24^.

It has been widely believed that photoreceptors utilize most glucose to generate lactate and only a small amount of pyruvate is metabolized through mitochondrial OXPHOS^3,15,18,25^. Recent studies have found that photoreceptors rely on glycolysis for the biogenesis of their outer segments and that genetic disruption of glycolysis results in photoreceptor degeneration^2,13,19,23^. However, Petit et al.^14^ recently proposed that aerobic glycolysis is not critical in photoreceptors but is rather a metabolic choice to maximize their function. The authors observed that the number and size of mitochondria in rod photoreceptors increased progressively to adapt to the inhibition of glycolysis after the deletion of HK2 in rods^14^. Exactly how photoreceptors balance aerobic glycolysis and mitochondrial OXPHOS to regulate their survival is still unclear. Here we have studied the long-term effect of selectively knocking down HK2 in rods on photoreceptor health and explore how the stressed retina undergoes metabolic remodeling to adapt to the inhibition of glycolysis in rods.

## Methods

### Animals

The animal studies were approved by The University of Sydney Animal Ethics and Biosafety Committees and performed in accordance with the Association of Ophthalmology and Vision Research (ARVO) statement for the use of animals in ophthalmology and vision research. Rhodopsin (RHO)-Cre mice were generated as described previously^26^. Mice that harbor LoxP sites at intron 3 and intron 10 of the HK2 gene were obtained from the European Mouse Mutant Archive (HK2-floxed mice, deposited by Dr Eija Pirinen). These mice have a C57BL/6J genetic background. We performed PCR using DNAs extracted from mouse tail samples to exclude the rd8 mutation before crossing RHO-Cre mice with HK2-floxed mice^27^. We crossed RHO-Cre mice with HK2-floxed mice to selectively knock down HK2 in rods, with age-matched HK2 wild-type (WT) mice serving as controls. As previous studies found limited retinal changes around 5 months after disruption of glycolysis^13,14,19^, we characterized changes in the retina in two groups of mice: young mice at 19~20 weeks and aged mice 40~41 weeks of age. We used 4 retinas from individual mice per group for Western blots on each polyvinylidene difluoride (PVDF) membrane, 4 eyes/group for immunohistochemistry (IHC) on frozen sections, 8 mice/group for electroretinography (ERG), optical coherence tomography (OCT) and 7–8 retinas/group for metabolic analysis, in the study as described below.

### IHC using frozen sections and retinal wholemounts

In brief, eyecups were dissected and fixed with 4% paraformaldehyde (PFA) for one hour and then transferred to phosphate-buffered solution (PBS) containing 30% sucrose at +4 °C for overnight. The fixed eyecups were embedded in optimal cutting temperature compound for frozen sectioning and IHC. Frozen sections were blocked by PBS containing 10% donkey serum and 1% Triton X100 at 4 °C for 2 h and then incubated with primary antibodies at 4 °C for overnight (Table 1). Sections were incubated with the corresponding secondary antibodies at room temperature for 4 h, counterstained with Hoechst and mounted for confocal fluorescent microscopy as previously described^28–30^.

**Table 1:** Listed antibodies used for Western blots (WB) and immunohistochemistry (IHC).

Antibody	Company and Cat#	Source	Dilution for WB	Dilution for IHC
Cre	Merck Millipore, MAB3120	Mouse	1:4000	1:500
Cone arrestin	Santa Cruz, AB15282	Rabbit	1:2000	–
HK1	Cell Signaling, CST#2024	Rabbit	1:2000	1:200
HK2	Cell Signaling, CST#2867	Rabbit	1:2000	1:200
HSP60	Cell Signaling, CST#12165	Rabbit	1:2000	1:200
IRBP	Abcam, ab101456	Rabbit	1:2000	1:200
OGDH	Sigma, HAP020347	Rabbit	–	1:200
OGDH	Thermo Scientific, PA5-28195	Rabbit	1:2000	–
PDHE1α	GeneTex, GTX104015	Rabbit	1:2000	1:200
PKM2	Cell Signaling, CST#4053	Rabbit	1:2000	1:200
p-PKM2^Tyr105^	Cell Signaling, CST#3827 S	Rabbit	1:1000	1:200
Recoverin	Merck Millipore, #AB5585	Rabbit	1:2000	1:200
VDAC	Cell Signaling, CST#4661	Rabbit	1:2000	1:200

*HK* hexokinase, HSP60 heat-shock protein 60, *IRBP* interphotoreceptor retinoid-binding protein, *OGDH* 2-oxoglutarate dehydrogenase, *PDHE1α* pyruvate dehydrogenase E1 subunit α, *PKM* pyruvate kinase muscle type, *p-PKM2*^*Tyr105*^ phosphorylated pyruvate kinase muscle type 2 at tyrosine residue 105, *VDAC* voltage-dependent-anion channel.

For immunostaining on retinal wholemounts, eye cups were fixed in 4% PFA for one hour and then stored in PBS at +4 °C. On the day of immunostaining, retinas were isolated, permeabilized, and incubated with primary antibodies against cone arrestin (1:500, Millipore#AB15282) and Cre (Merck Millipore, MAB3120, 1:500) at 4 °C for overnight. After incubation with the corresponding secondary antibodies at room temperature for 4 h, sample were counterstained with Hoechst and flat-mounted for confocal fluorescent microscopy^28–30^.

### Western blots

Proteins were extracted from retinas and their concentrations were measured by bicinchoninic acid assays. Twenty microgram proteins were loaded to the SDS-polyacrylamide gel electrophoresis and then transferred to PVDF membranes. The PVDF membranes were incubated with primary antibodies (Table 1) at 4 °C for overnight followed by an incubation with the corresponding secondary antibodies at room temperature for 4 h. Protein bands were imaged by G: Box BioImaging systems (Syngene, Frederick, MD, USA) and quantified by GeneTools image scanning and analysis package. The expression of proteins was normalized to the expression of α/β tubulin, which served as loading controls.

### ERG and OCT

ERG was performed using the Phoenix Ganzfeld ERG system (Phoenix Research Laboratories Inc., Pleasanton, USA). In brief, mice were dark-adapted overnight and anesthetized with 48 mg/kg ketamine and 0.6 mg/kg medetomidine. Pupils were dilated with tropicamide and phenylephrine and eye gel applied to protect the cornea. Two-needle electrodes were inserted under the skin between the head and ears and the tail, respectively. Electroretinographic responses were recorded over a range of stimulus intensities under 505 nm wavelength green light. The a-wave amplitude was measured from the baseline to the trough of the a-wave response and the b-wave amplitude was measured from the trough of the a-wave to the peak of the b-wave.

OCT scanning was conducted using the Phoenix mouse OCT2 system (Phoenix Research Laboratories Inc., Pleasanton, USA). In brief, a live fundus image was used to guide OCT scanning in the central retina across and just above the optic nerve head and in the peripheral retina in each eye. The thickness of the outer nuclear layer (ONL) and photoreceptor inner and outer segments was measured using the software installed in the system.

### ^13^C-glucose labeling, gas and liquid chromatography-mass spectrometry

Mice received intraperitoneal injection of 500 mg/kg of uniformly labeled-glucose (^13^C_6_-glucose) and were euthanized with cervical dislocation 60 min after injection. Retinas were collected within one minute and snap frozen in liquid nitrogen. Samples were stored at −80 °C for analysis of ^13^C-glucose-derived metabolites in glycolysis and the TCA cycle using mass spectrometry as described previously^12,31^. In brief, Retinas were homogenized with 20 μl of 80% methanol, placed on dry ice for 30 min and centrifuged for 15 min at 15,000 rpm, 4 °C. Supernatants were transferred to a glass insert and mixed with 0.1 mM myristic-d_27_ acid as an internal standard and then dried under vacuum at 4 °C.

For gas chromatography-mass spectrometry (GC-MS), the dried metabolites were derivatized with methoxyamine hydrochloride N-tert-butyldimethylsilyl- N-methyltrifluoroacetamide before analyzed by an 7890/5977B GC/MS system (Agilent Technologies) with an Agilent DB-5MS column as previously described^12^. LC-MS was performed with a Shimadzu LC Nexera X2 UHPLC coupled with a QTRAP 5500 LC MS (AB Sciex) as previously described^12,32^. An ACQUITY UPLC BEH Amide analytic column (2.1 × 50 mm, 1.7 μm, Waters) was used for chromatographic separation. The mobile phase was (A) water with 10 mM ammonium acetate (pH 8.9) and (B) acetonitrile/water (95/5) with 10 mM ammonium acetate (pH 8.2). The gradient elution was 95–61% B in 6 min, 61–44% B at 8 min, 61–27% B at 8.2 min, and 27–95% B at 9 min. The column was equilibrated with 95% B at the end of each run. The collision gas was N2. The ion source conditions in positive and negative mode were: curtain gas (CUR) = 25 psi, collision gas (CAD) = high, ion spray voltage (IS) = 3800/- 3800 V, temperature (TEM) = 500 °C, ion source gas 1 (GS1) = 50 psi, and ion source gas 2 (GS2) = 40 psi. Each metabolite was tuned with standards for optimal transitions and ^13^C-nicotinic acid (Toronto Research Chemicals) was used as the internal standard. The extracted MRM peaks were integrated using MultiQuant 3.0.2 software (AB Sciex).

### Statistical analysis

Results were expressed as mean ± SEM. All statistical analyses were conducted using the GraphPad Prism 7.0 statistics software released by GraphPad Software, Inc. The statistical significance of normally distributed or non-normally distributed data was evaluated using the two-tailed unpaired Student’s test or Mann–Whitney test and differences were considered significant at *P < 0.05, **P < 0.01, and ***P < 0.001, respectively. We also used a power calculation tool^33^ deposited at https://psychologie.hhu.de/arbeitsgruppen/allgemeine-psychologie-und-arbeitspsychologie/gpower.html to estimate the sample sizes for achieving statistical differences between wild-type control and HK2 knockdown mice in Western blot analysis and ERG studies.

## Results

### Crossing RHO-Cre mice with HK2-floxed mice led to deletion of HK2 in the retina

**Fig. 1:**
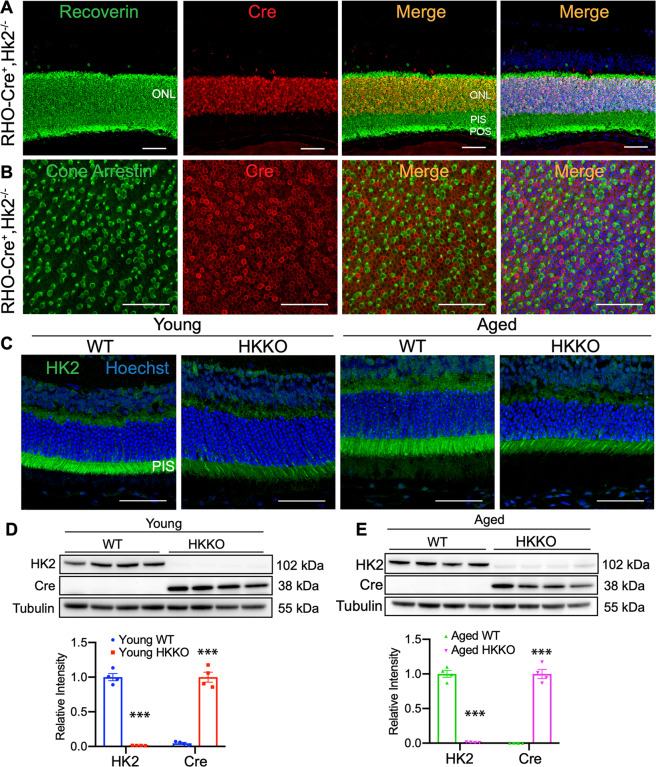
Deletion of HK2 in RHO-Cre mice crossed with HK2-floxed mice. **A** Recoverin, a marker of rod receptors, was expressed in the outer nuclear layer (ONL) and photoreceptor inner and outer segments (PIS and POS). Cre recombinase was exclusively expressed in cell nuclei positive for recoverin in the ONL in RHO-Cre mice. **B** Double label IHC in retinal wholemounts from RHO-Cre mice indicated that Cre recombinase was not expressed in cone photoreceptors. **C**–**E** Knockdown of HK2 in RHO-Cre mice crossed with HK-floxed mice (hereafter called HKKO mice). **C** IHC indicated that HK2 was dramatically reduced in photoreceptor inner segments (PIS) in HKKO mice compared with age-matched wild-type (WT) controls. Scale bars: 50μm in (**A**–**C**). **D**, **E** Western blots indicated that the expression of HK2 was reduced by ~95% in the retina of HKKO mice compared with age-matched WT controls. *N* = 4–8/group. ***p < 0.001, analyzed by un-paired *t*-test.

Recoverin, a marker of rod receptors, was predominantly expressed in the ONL and photoreceptor inner and outer segments (PIS and POS) (Fig. 1A). Double label IHC indicated that Cre recombinase was exclusively expressed in cell nuclei positive for recoverin in the ONL in RHO-Cre mice (Fig. 1A). Double label IHC for cone arrestin and Cre recombinase using flatmounted retinas confirmed that cone photoreceptors did not express Cre recombinase (Fig. 1B). These results confirmed the rod-specific gene targeting in RHO-Cre mice as reported previously^26^.

The deletion of HK2 was confirmed by IHC and Western blots using retinas from RHO-Cre mice crossed with HK2-floxed mice (hereafter referred to as HKKO mice) and age-matched WT controls. HK2 was predominately expressed in photoreceptor inner segments (PIS) in the normal retina. A dramatic reduction of HK2 expression was observed in PIS in young and aged HKKO mice (Fig. 1C)). Western blots found that HK2 was reduced by ~95% in young and aged HKKO mice compared with age-matched WT controls (Fig. 1D, E).

### Deletion of HK2 in rods led to reduced expression of photoreceptor-specific proteins

**Fig. 2:**
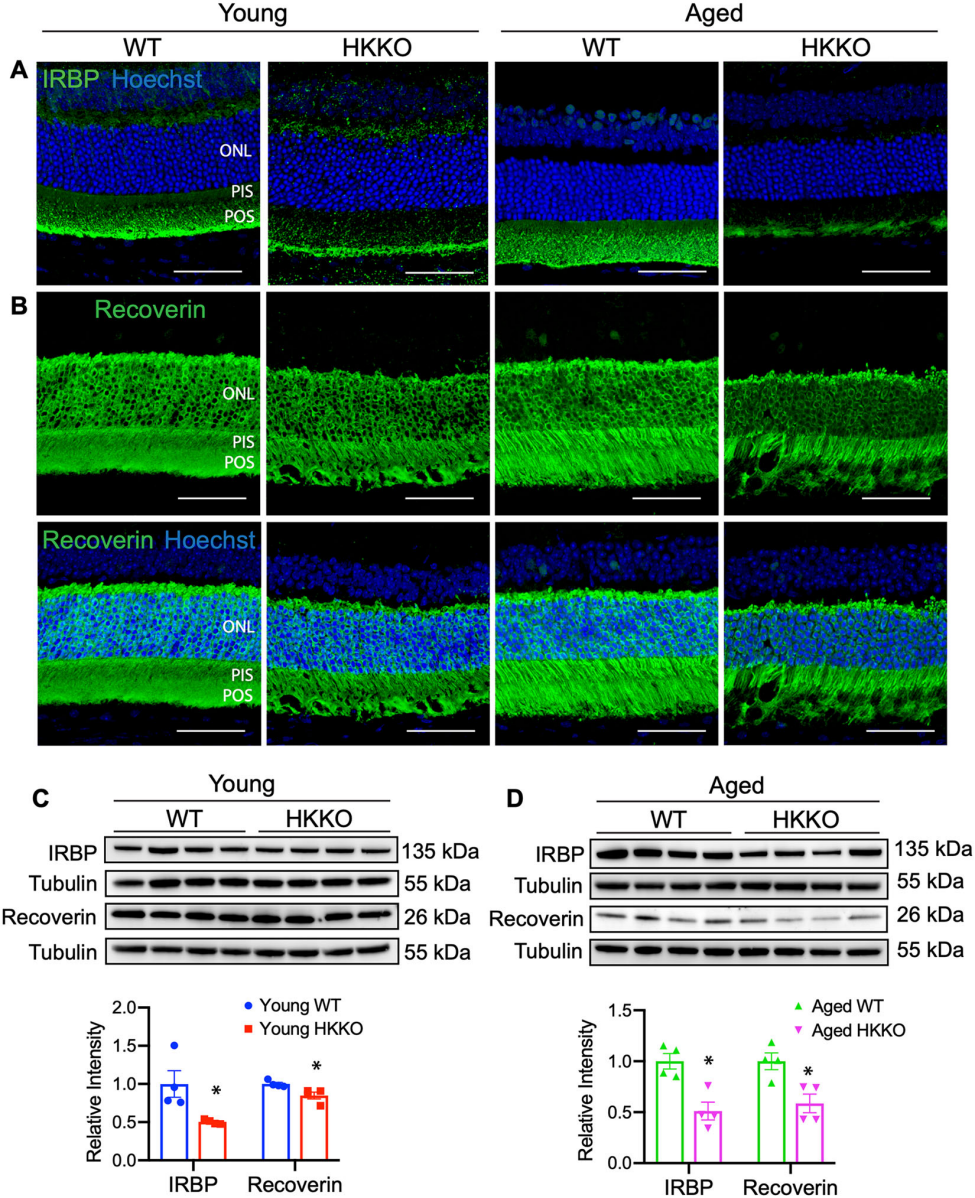
Knockdown of HK2 in rods led to reduced expression of photoreceptor-specific protein. **A**, **B** Immunostaining for interphotoreceptor retinoid-binding protein (IRBP) and recoverin in retinal sections from HKKO mice and age-matched WT controls. Scale bar: 50 μm in (**A**) and (**B**). **C**, **D** Western blots indicated that the expression of IRBP and recoverin was significantly reduced in HKKO mice compared with age-matched WT controls. *N* = 4/group. *p < 0.05, analyzed by un-paired *t* test.

We performed IHC and Western blots to study changes in photoreceptor-specific proteins including interphotoreceptor retinoid-binding protein (IRBP) and recoverin (Fig. 2). IRBP plays an important role in retinoid transport between photoreceptors and retinal pigment epithelial (RPE) cells^34^. We previously found that loss of IRBP is a sensitive marker of photoreceptor degeneration^35,36^. Recoverin is a calcium-binding protein predominantly expressed by photoreceptors that functions as a calcium sensor to regulate rhodopsin phosphorylation during phototransduction^37^. We found that IRBP was expressed in POS in the normal retina but was reduced in young and aged HKKO mice (Fig. 2A). Recoverin was expressed in the ONL, PIS, and POS in the normal retina (Fig. 2B). Reduced expression of recoverin was observed in the ONL but less obvious in PIS and POS in young compared with aged HKKO mice (Fig. 2B). The deletion of HK2 led to severe disruption of POS in aged but not in young HKKO mice (Fig. 2B). Western blots indicated that IRBP expression was decreased by ~50% in the retinas of young and aged HKKO mice while recoverin was reduced by 10~15% in young HKKO mice and 30~40% in aged HKKO mice compared with age-matched WT controls (Figs. 2C, D).

### Deletion of HK2 in rods led to age-related photoreceptor degeneration and impaired retinal function

**Fig. 3:**
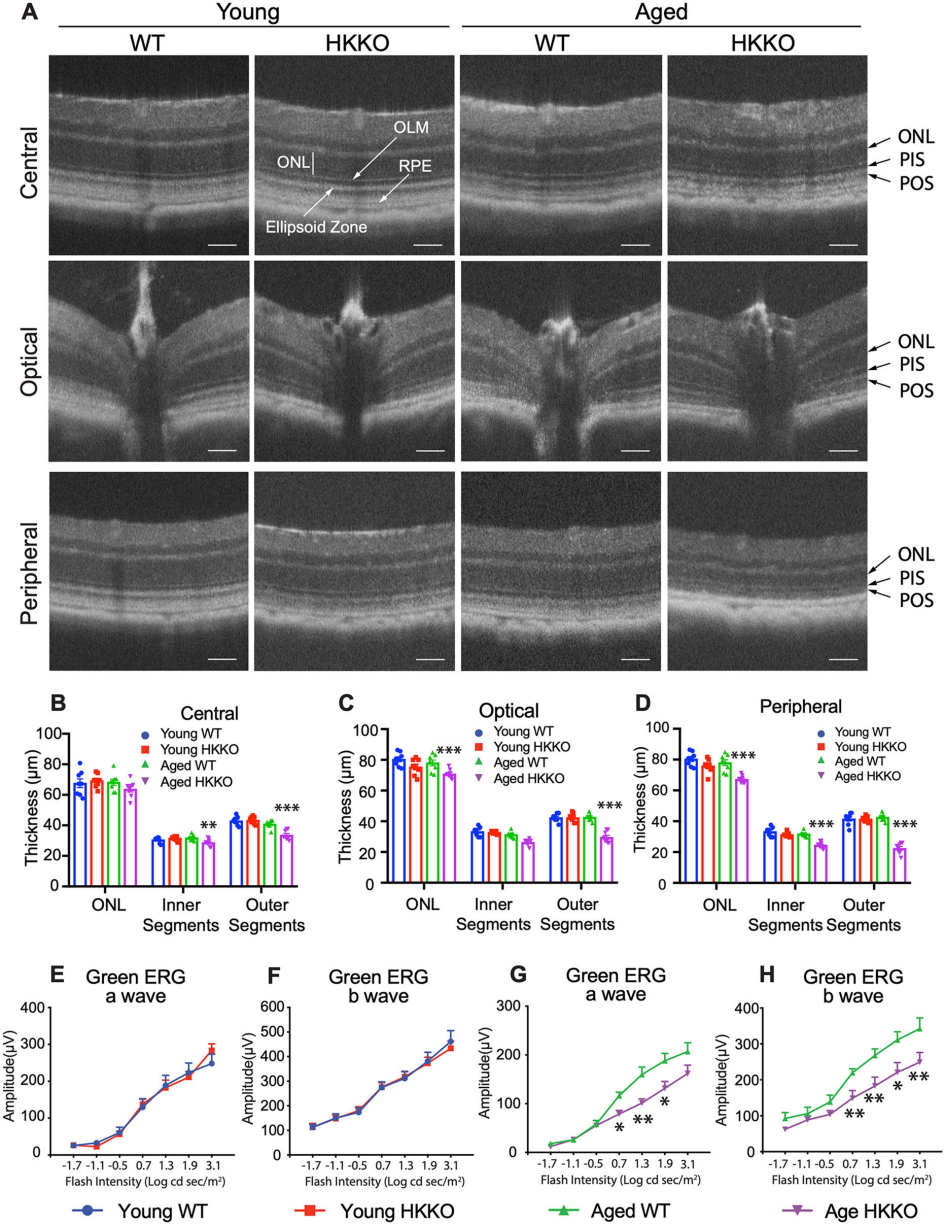
Knockdown of HK2 in rods led to aged-related photoreceptor degeneration and impaired retinal function. **A** Image-guided OCT was performed on the central retina in areas just above the optic nerve head and across the optic nerve head as well as on the peripheral retina. ONL outer nuclear layer, OLM outer limiting membrane, PIS photoreceptor inner segment, referring to the vertical length from the OLM to the outer border of the ellipsoid zone, POS photoreceptor outer segment, referring to the vertical length from the outer border of the ellipsoid zone to the apical side of the retinal pigment epithelium (RPE). **B**–**D** Changes in thickness of the ONL, PIS, and POS in the three regions in young and aged mice after knocking down HK2 in rods. *P < 0.05, **P < 0.01, *n* = 8/group. **E**–**H** Scotopic ERG measured under stimulation with a range of intensities of green (505 nm) light in young and aged HKKO mice and age-matched controls. **E**, **F** There were no significant changes in the amplitudes of a and b waves in young HKKO mice compared with age-matched WT controls. **G**, **H** The amplitudes of both a and b waves were significantly reduced in aged HKKO mice compared with age-matched WT controls. *P < 0.05, **P < 0.01, *n* = 8/group.

We performed OCT to study changes in the outer retinal structure (Fig. 3). Quantitative analysis of OCT images indicated that there were no significant changes in the thickness of the ONL, PIS, and POS in young HKKO mice compared with age-matched WT controls but disruption of the outer retinal structure was observed in aged HKKO mice (Fig. 3B–D), as evidenced by a mean reduction of 10–15% of the thickness of the ONL, 7–10% of the PIS and 20–25% reduction of POS in the central retina compared with 20% reduction of PIS and 50% reduction of POS in the peripheral retina (Fig. 3B–D).

Scotopic ERG was performed to study changes in retinal function (Fig. 3E–H). The scotopic ERG was recorded under 505 nm wavelength green light, which stimulates rod and M cone photoreceptors^38^. The amplitudes of a and b waves remained unaffected in young HKKO mice (Fig. 3E, F). However, significant reductions in the amplitudes of the a and b waves were observed in aged HKKO mice (Fig. 3G, H). These results indicate that, consistent with our IHC studies above, the deletion of HK2 leads to age-related photoreceptor degeneration.

### Deletion of HK2 in rods led to upregulation of retinal mitochondrial stress proteins

**Fig. 4:**
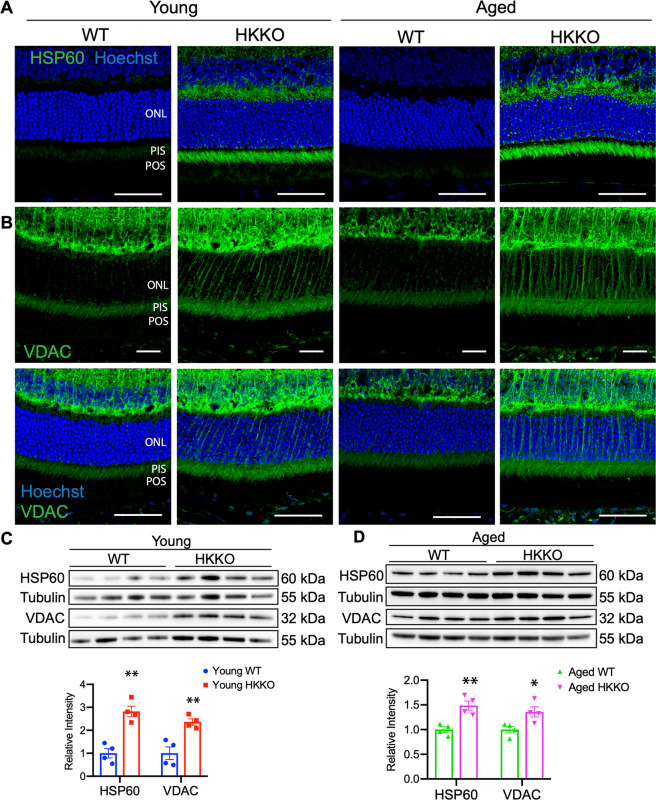
Knockdown of HK2 led to upregulation of mitochondrial proteins including heat-shock protein 60 (HSP60) and voltage-dependent-anion channel (VDAC). **A**, **B** Immunostaining for HSP60 and VDAC in retinal sections from HKKO mice and WT controls. **A** Enhanced expression of HSP60 was observed in the photoreceptor inner segments (PIS) and the outer plexiform layer (OPL) in HKKO mice. **B** Increased expression of VDAC was observed in the outer retina of HKKO mice. The upper panel images are immunostaining for VDAC without Hoechst nuclear counterstaining. Scale bar: 50 μm in (**A**, **B**). **C**, **D** Western blots indicated that the expression of HSP60 and VDAC was significantly increased in HKKO mice compared with age-matched WT controls. *p < 0.05 and **p < 0.01, *n* = 4/group.

We performed IHC and Western blots to study changes in mitochondrial stress markers including heat-shock protein (HSP) 60 and VDAC (Fig. 4). HSP60 is a chaperon protein that facilitates protein folding and maintains mitochondrial DNA replication when mitochondria are stressed^39–41^. VDAC, located to the outer membrane of mitochondria, is responsible for regulating the transport of ions and metabolites in and out of mitochondria^42^. We found that HSP60 and VDAC were weakly expressed in PIS in the normal retina but both were increased in PIS and the outer plexiform layer (OPL) in young and aged HKKO mice (Fig. 4A, B). Western blots confirmed that the expression of HSP60 and VDAC was significantly increased in the retina of HKKO mice compared with age-matched WT controls (Fig. 4C, D).

### Loss of HK2 in rods led to upregulation of HK1 and TCA cycle enzymes and phosphorylation of PKM2 at tyrosine residue 105 (p-PKM2^Tyr105^)

**Fig. 5:**
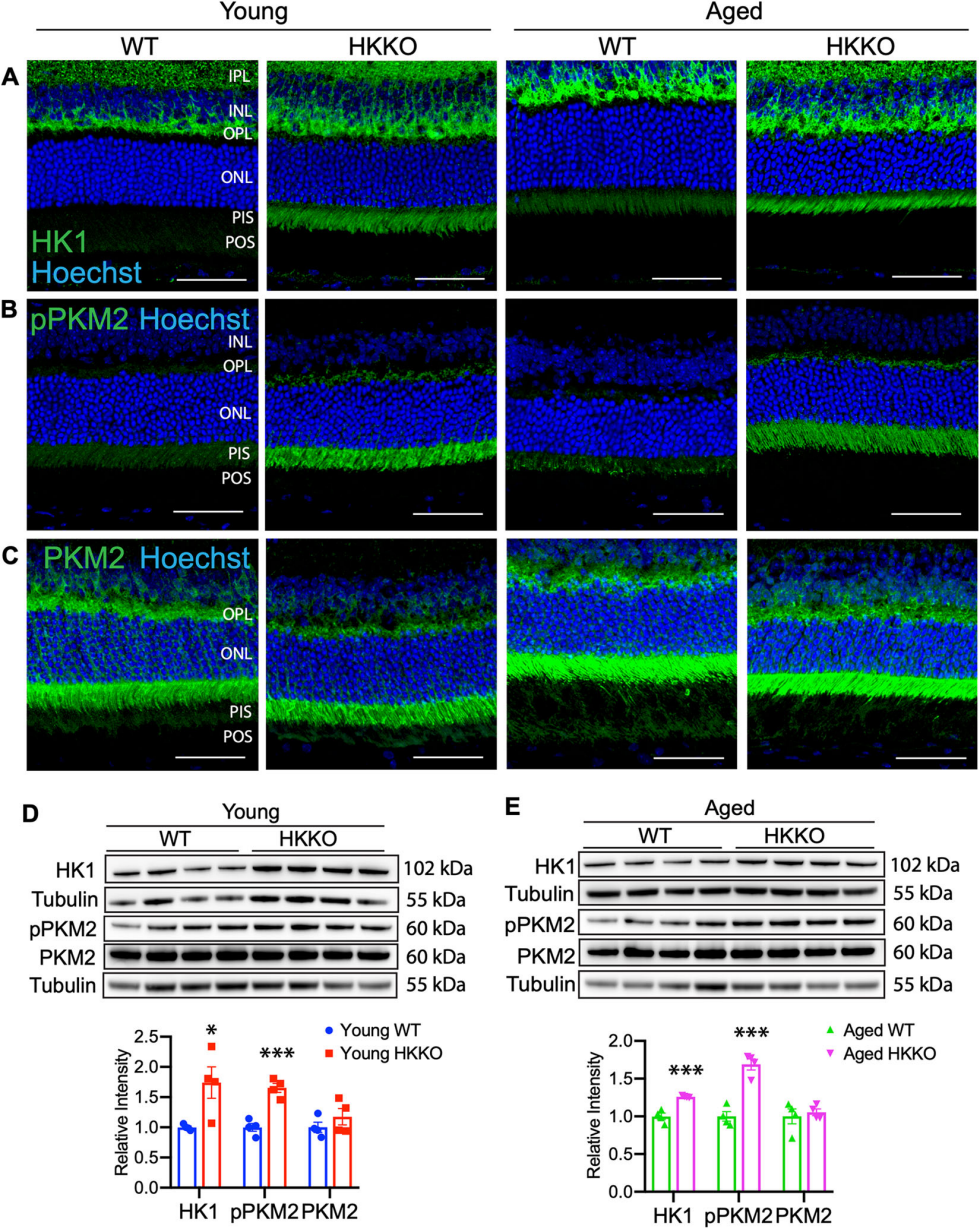
Loss of HK2 in rods led to increased expression of HK1 and phosphorylation of PKM2 at tyrosine residue 105 (p-PKM2). **A**–**C** Immunostaining for HK1, pPKM2, and PKM2 in retinal sections from HKKO mice and age-matched WT controls. Increased expression of HK1 (**A**) and pPKM2 (**B**) were observed in photoreceptor inner segments (PIS) in retinas of HKKO mice. Scale bar: 50 μm in (**A**–**C**). **D**, **E** Western blots indicated that the expression of HK1 and pPKM2 was significantly increased while the level of PKM2 expression remained unchanged in HKKO mice compared with age-matched WT controls. *p < 0.05 and ***p < 0.001, *n* = 4/group.

We performed IHC and Western blots to study changes in glycolytic enzymes including HK1 and PKM2 (Fig. 5). HK1 was strongly expressed in OPL and the inner plexiform layer (IPL) of the normal retina but only weakly expressed in PIS (Fig. 5A). The deletion of HK2 in rods did not change the patterns of HK1 expression in the OPL and IPL but increased HK1 expression was observed in PIS (Fig. 5A). Deletion of HK2 in rods increased expression of p-PKM2^Tyr105^ in PIS (Fig. 5B) while the total form of PKM2 remained relatively unchanged in each age group (Fig. 5C). Western blots found that knock down of HK2 in rods significantly increased the expression of HK1, p-PKM2^Tyr105^, and the ratio of p-PKM2^Tyr105^/PKM2, while the total PKM2 remained unchanged compared with age-matched controls (Fig. 5D, E).

**Fig. 6:**
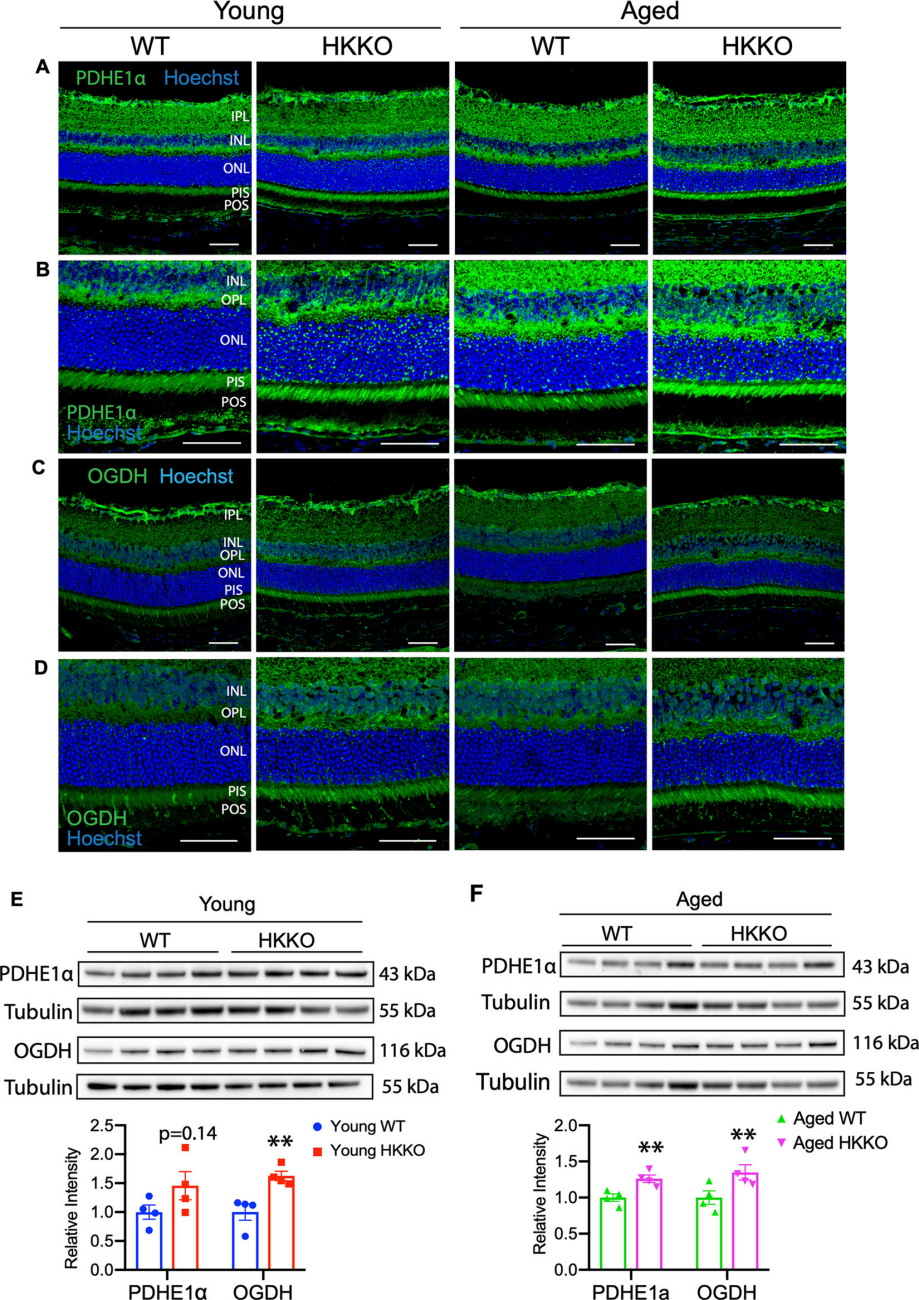
Knocking down HK2 led to upregulation of enzymes in the TCA cycle. **A**–**D** Immunostaining for enzymes in the TCA cycle including pyruvate dehydrogenase E1α (PDHE1α, **A** and **B**) and oxoglutarate dehydrogenase (OGDH, **C** and **D**) in retinal sections from HKKO mice and age-matched WT controls. Loss of HK2 in rods led to increased expression of PDHE1α (**A**, **B**) and OGDH (**C**, **D**) in the photoreceptor inner segments (PIS). The panel of images in (**B**) and (**D**) are higher-power images of areas in (**A**) and (**C**) respectively. Scale bar: 50 μm in (**A**–**D**). **E**, **F** Western blots indicated that the expression of PDHE1α (**C**) and OGDH (**D**) was significantly increased in HKKO mice compared with age-matched WT controls. *p < 0.05 and **p < 0.01, *n* = 4/group.

We next studied changes in two rate-limiting enzymes in the TCA cycle, PDHE1α and oxoglutarate dehydrogenase (OGDH), after deletion of HK2 in rods (Fig. 6). PDHE1α, the first enzyme in the TCA cycle, is responsible for converting pyruvate to acetyl-CoA for mitochondrial OXPHOS^43^. Acetyl-CoA enters into the TCA cycle and is catalyzed to citrate and αKG^44^. OGDH catalyses αKG to produce succinyl Co A that is then converted to succinate to be used by the TCA cycle^45^. We found that PDHE1α was expressed in PIS, OPL, IPL, and the ganglion cell layer (GCL) in the normal retina. The deletion of HK2 in rods led to increased expression of PDHE1α in PIS (Fig. 6A, B). Similarly, the normal retina expressed little OGDH, deletion of HK2 in rods led to increased expression of OGDH in PIS, OPL, IPL, and GCL, with the strongest immunoreactivity being observed in PIS (Fig. 6C, D). Western blots confirmed that the deletion of HK2 in rods significantly increased the expression of PDHE1α and OGDH (Fig. 6E, F). These collective results suggest that the deletion of HK2 in rods leads to retinal metabolic remodeling.

### Deletion of HK2 in rods led to reduced glycolysis and enhanced mitochondrial OXPHOS in the retina

**Fig. 7:**
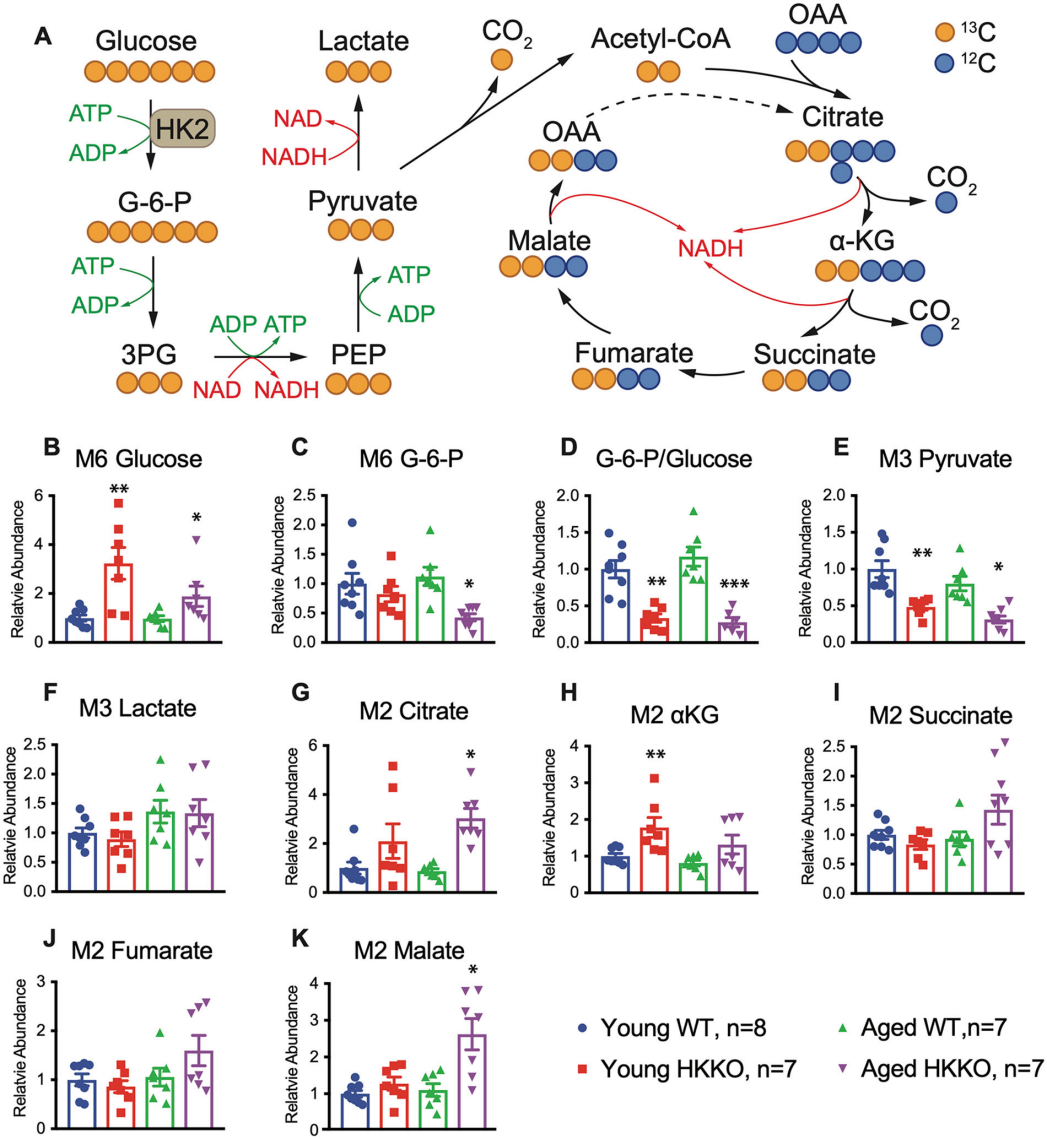
Knockdown of HK2 in rods led to retinal metabolic remodeling. **A** Schematic diagram of labeled metabolites derived from ^13^C-glucose in glycolysis and the TCA cycle. **B**–**P** Changes in the relative abundance of ^13^C-glucose (**B**) and its derivatives in glycolysis (**C**–**F**) and the TCA cycle (**G**–**K**) in HKKO mice compared with age-matched WT controls. The value of each metabolite was normalized to its abundance in young WT controls. G-6-P = glucose-6-phosphate (G-6-P), 3PG glyceraldehyde-3-phosphate, PEP phosphoenolpyruvate, OAA oxaloacetate (OAA), αKG Ketoglutarate. *p < 0.05 and **p < 0.01, *n* = 7~8/group.

We used in vivo labeling of retinas with ^13^C-glucose to study changes in glycolysis and the TCA cycle after deletion of HK2 in rod (Fig. 7A). Deletion of HK2 in rods led to the retention of ^13^C-glucose (M6 glucose) (Fig. 7B) and decreased production of M6 glucose-6-phosphate (G-6-P) but only aged HKKO mice had significantly reduced M6 G-6-P compared with age-matched WT controls (Fig. 7C). The ratios of M6 G-6-P/M6 glucose were significantly reduced in the retina from young and aged HKKO mice (Fig. 7D). We found that M3 pyruvate, the end product of glycolysis, was also significantly reduced (Fig. 7E) while M3 lactate remained unchanged in young and aged HKKO mice (Fig. 7F). These results indicate that loss of HK2 in rods led to inhibition of retinal glycolysis.

We next studied changes in ^13^C-glucose-derived metabolites in the TCA cycle including M2 citrate, α-ketoglutarate, succinate, fumarate, and malate (Fig. 7G–K). Loss of HK2 in rods led to increased production of metabolites in the TCA cycle, with more obvious alterations being observed in aged mice than in young mice (Fig. 7G–K). These results indicate that mitochondrial OXPHOS is enhanced in stressed retinas to adapt to the reduced glycolysis resulting from the deletion of HK2 in rods.

## Discussion

The Warburg effect refers to modified glucose metabolism where cancer cells and photoreceptors tend to metabolize most glucose through aerobic glycolysis^17,18,46^. Despite this, photoreceptors possess abundant mitochondria and enzymes for OXPHOS^15,25^. A better understanding of the contributions of aerobic glycolysis and mitochondrial OXPHOX to photoreceptor health is of biological and therapeutic interest. We used a rod photoreceptor-specific approach here to study the long-term effects of selectively knocking down HK2 in rods on retinal health and metabolic remodeling. We found that knocking down HK2 led to photoreceptor degeneration, which was more marked in older animals, as suggested by reduced expression of photoreceptor-specific proteins, changes in the outer retinal structure and impaired retinal function.

We found that knocking down HK2 in rods inhibited retinal glycolysis and led to chronic but not rapid photoreceptor degeneration. IHC and western blots found that the expression of HK2 was reduced by at least 95% in young (19–20 weeks of age) and aged (40–41 weeks of age) HKKO mice. However, OCT and ERG measurements indicated that the retinal structure and function remained unchanged in young HKKO mice, although Western blots revealed reduced expression of photoreceptor-specific proteins including IRBP and recoverin. Others have similarly observed that the deletion of HK2^14,19^ or PKM2^13^ in rods resulted in no or mild changes in photoreceptors in mice around 5 months of age.

Photoreceptors express low levels of HK1 in their inner segments in the normal retina. We found that the deletion of HK2 in rods led to increased expression of HK1 in PIS, consistent with reports that deletion of HK2 or PKM2 in rods led to compensatory upregulation of glycolytic enzymes, including HK1 and PKM1^10,13,19,23^. These results indicated that stressed photoreceptors may express alterative isoforms of enzymes to conduct glycolysis after deletion of HK2 or PKM2 in rods^10,13,19,23^. Analyses of metabolites in the glycolytic pathway found retention of ^13^C-labeled M6 glucose and reduced production of ^13^C-glucose derived M3 pyruvate after the loss of HK2 in rods (Fig. 7). HK1/2 catalyzes the generation of G-6-P from glucose. The significant reduction of ^13^C-glucose-derived G-6-P in aged but not young HKKO mice suggest that HK1 may temporarily compensate for the reduced HK activity resulting from loss of HK2. However, the significant retention of ^13^C-glucose and reduced downstream labeling of M3 pyruvate indicate that the reactive upregulation of HK1 did not fully compensate for the lost HK2 activity in rods. Further research is warranted to compare the HK activities between normal and HKK2 retinas with different concentrations of substrate and steady-state labeling under continuous in vivo infusion of ^13^C-glucose.

**Fig. 8:**
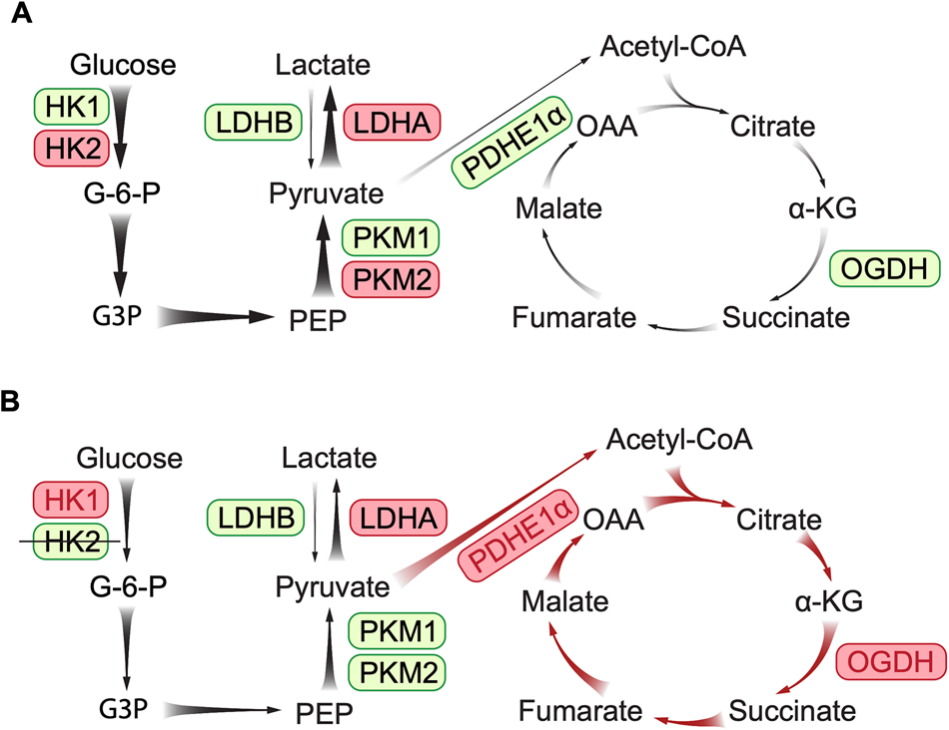
Proposed mechanism of metabolic remodeling after inhibiting aerobic glycolysis resulted from the deletion of HK2 in rods. **A** Glycolysis and mitochondrial oxidative phosphorylation (OXPHOS) in the normal retina. Glucose is mainly metabolized to pyruvate through glycolysis using enzymes including HK2, PKM2 and lactate dehydrogenase A (LDHA). A small amount of pyruvate is catalyzed by PDHE1α to produce acetyl-CoA for mitochondria OXPHOS in the TCA cycle. **B** Retinal metabolic remodeling after deletion of HK2 in rods. Loss of HK2 in rods leads to enhanced activities of HK1 and upregulation of pPKM2. The resultant production of pyruvate is mainly used for mitochondrial OXPHOS to adapt to the inhibition of aerobic glycolysis. The enzymes highlighted in red and the reactions indicated by thicker arrows represent the predominated enzymes and metabolic activities in normal and stressed retinas, while the enzymes highlighted in green and the reactions indicated by thinner arrows represent less-active enzymes and metabolic activities in each condition.

We found that knocking down HK2 in rods led to upregulation of the mitochondrial stress proteins HSP60 and VDAC. HSP60 is a mitochondrial chaperonin responsible for the transportation and refolding of proteins from the cytoplasm into the mitochondrial matrix^47^. VDAC is located to the outer membrane of mitochondria and responsible for governing the permeability of the outer mitochondrial membrane to ions and metabolites^48–50^. It pumps ATP out of mitochondria to the cytosol and transports pyruvate, adenosine diphosphate (ADP), and phosphate from the cytosol to mitochondria when it is open^48–50^. HK2 can bind to VDAC to regulate the transport of metabolites for ATP production to fuel the first step of glycolysis^51^. Loss of HK2 in rods would open the VDAC channel, as a result, metabolites such as pyruvate, ADP, and phosphate enter into mitochondria to promote OXPHOS^42,52,53^. We found that the deletion of HK2 in rods increased expression of the TCA enzymes PEHE1α and OGDH, along with increased production of ^13^C-glucose-derived metabolites by the TCA cycle. Our findings are consistent with a previous observation that rod photoreceptors increased the number and size of their mitochondria to adapt to the inhibited glycolysis after the deletion of HK2 in rods^14^. We also found that the deletion of HK2 in rods led to increased expression of p-PKM2^Tyr105^ while the total PKM2 remained unchanged. Previous studies found that phosphorylation of PKM2 at Tyr105 in cancer cells inhibits the formation of active, tetrameric PKM2 and that mutant PKM2 made cancer cells more dependent on OXPHOS for cell metabolism and proliferation^54,55^. These collective results support a mechanism that the stressed retina enhances mitochondrial OXPHOS to adapt to the inhibition of aerobic glycolysis after the deletion of HK2 in rods (Fig. 8).

We found that inhibition of glycolysis led to photoreceptor degeneration that was more obvious in older animals. On the one hand, metabolic reprogramming from glycolysis to mitochondrial OXPHOS may partially reduce the metabolic stress caused deletion of HK2, on the other, excessive mitochondrial OXPHOS promotes the generation of reactive oxygen species (ROS), which can finally lead to mitochondrial dysfunction and photoreceptor degeneration. Further research is warranted to study changes in the production of ROS in situ using fluorescent ROS signaling sensors^56,57^ after selectively knocking down HK2 in rod photoreceptors.

We found that the accumulation of metabolites of the TCA cycle was more obvious in aged mice than young mice after the deletion of HK2 in rods. Recent studies indicate that the accumulation of metabolites in the TCA cycle is a sign of mitochondrial stress and dysfunction^58–60^. Our Western blots found a more significant reduction in expression of recoverin in aged mice than young mice after the deletion of HK2. Significant shortening of the ONL, PIS, and POS was observed in aged but not in young HKKO mice. Changes in the retinal outer structure were also supported by the ERG results that aged HKKO mice had significant reductions in the amplitudes of both a and b waves but these changes were not found in young HKKO mice. These collective findings suggest that the deletion of HK2 in rods eventually leads to age-related photoreceptor degeneration.

In summary, our study indicates that HK2-mediated aerobic glycolysis in rods is indispensable for the maintenance of photoreceptor structure and function. Loss of HK2 in rods leads to enhanced expression of HK1 but the compensatory upregulation of HK1 does not fully circumvent the deficiency in glycolysis resulting from the deletion of HK2. Long-term inhibition of glycolysis leads to photoreceptor degeneration.
